# Multistage Cyclic Dielectrophoresis for High-Resolution Sorting of Submicron Particles

**DOI:** 10.3390/mi16040404

**Published:** 2025-03-29

**Authors:** Wenshen Luo, Chaowen Zheng, Cuimin Sun, Zekun Li, Hui You

**Affiliations:** 1 College of Mechanics, Guangxi University, 100 East University Road, Nanning 530004, China; 2011301035@st.gxu.edu.cn (W.L.); lizekun1666@163.com (Z.L.); 2 School of Mechanical and Electrical Engineering, Beihai Vocational College, 48 Tibet Road, Beihai 536009, China; cwzheng95@163.com; 3 College of Computer and Electronic Information, Guangxi University, 100 East University Road, Nanning 530004, China; cmsun@gxu.edu.cn

**Keywords:** submicrometer particle, dielectrophoresis, multistage cycle, precise screening

## Abstract

The precise preparation and application of nanomicrospheres is currently an emerging research hotspot in the cutting-edge cross-disciplines. As an important functional material, nanosized microspheres show a broad application prospect in biomedicine, chemical engineering, materials science, and other fields. However, microspheres with good monodispersity are still facing technical bottlenecks, such as complicated preparation process and high cost. In this study, a multistage cyclic dielectrophoresis (MC-DEP) technique is innovatively proposed to successfully realize the high-resolution sorting of submicron microspheres. A dielectrophoresis chip adopts a unique electrode design, in which the electrodes are arranged at the top and bottom of the microchannel at the same time. This symmetric electrode structure effectively eliminates the difference in the distribution of dielectrophoretic force in the perpendicular direction and ensures the homogeneity of the initial state of particle sorting. Three pairs of focusing electrodes are in the front section of the microchannel for preaggregation of the microspheres, and the deflection electrodes in the back section are to realize particle size sorting. After this, the upper and lower limits of particle size are limited by multiple cycles of sorting. The multistage cyclic sorting increases the stability of particle deflection under dielectrophoretic forces and reduces the error perturbation caused by the fluid environment. The experimental results show that the multistage cycling sorting scheme significantly improves the monodispersity of the microspheres, and the coefficient of variation of the particle size is significantly reduced from the initial 12.3% to 5.4% after three cycles of sorting, which fully verifies the superior performance of this technology.

## 1. Introduction

Submicron microspheres are tiny spheres with a particle size between 100 nm and 1 µm [1], which have a wide range of applications in the fields of medicine, biology, materials, and optics. In the biomedical field, submicron microspheres can be designed as drug delivery systems to achieve precise drug delivery to target tissues or cells [2,3]. By regulating the size, morphology, and surface properties of microspheres, the bioavailability of drugs can be significantly enhanced, while reducing their toxic side effects. Submicron microspheres also play an important role in chemical and materials science research, such as for the preparation of coatings and composites with special properties [4]. In the field of optics, submicron microspheres show unique application value, which can be utilized as microlenses, micro-optical elements, and photonics devices, thus expanding the functions of optical imaging and sensing [5]. In addition, submicron microspheres with excellent monodispersity are key materials for constructing photonic crystals [6]. With the rapid development of micro and nanotechnology, the application of submicron microspheres on the micro and nanoscale is becoming more and more widespread. Therefore, the precise control of microsphere particle size and the homogeneity of the particle size distribution become the core of the preparation technology. For example, in the preparation of photonic crystals with structural color, the application of sub-micron microspheres with good monodispersity is necessary. Furthermore, the typical particle size of the sub-micron microspheres range is about 200 nm. Due to their optical properties, photonic crystals are extremely sensitive to the uniformity of the microsphere particle size, the coefficient of variation of the microsphere particle size is required to be controlled to less than 5%. Furthermore, the smaller the coefficient of variation is, the better the quality of photonic crystals is [7].

In order to obtain high homogeneity microspheres suitable for specific fields, the microsphere size must be strictly regulated. Microfluidic technology shows great potential in the field of microsphere separation due to its advantages of low-power continuous operation, low-cost fabrication, and precise manipulation of micrometer-scale structures. In addition, most microfluidic techniques are non-contaminating and non-damaging to the sample. A variety of microfluidic separation techniques have been developed, including elastic inertial focusing [8,9], anisotropic microfluidic barriers [10], surface acoustic wave separation [11], microfluidic centrifugation [12], and coupled electric-acoustic-field separation [13]. However, despite the fact that microfluidics has shown its applicability in submicron particle separation. Contamination-free, high-efficiency, high-resolution sorting of submicron microspheres is still a major challenge. These microfluidic techniques are difficult to achieve the effective separation of particles with size differences of 50 nm, or less than 10%. Apart from that, most of the microfluidic technologies do not have high efficiency.

Dielectrophoresis, as a motion phenomenon of neutral particles in a non-uniform electric field, allows for non-contact manipulation of particles through dielectrophoretic forces, such as aggregation [14], sorting [15], and rotation [16]. Due to its active manipulation characteristics and flexibility, dielectrophoresis has significant advantages in realizing the high-precision sorting of microspheres. However, the current research on the sorting of submicron microspheres by dielectrophoresis is relatively limited, mainly due to the fact that the manipulation of submicron microspheres requires stronger electric field strengths and higher field gradients [17], which not only requires stronger electrical signal inputs, but more precise electrode machining to achieve smaller electrode spacing. In addition, submicron particles are susceptible to disturbances, such as Brownian motion [18], AC osmotic flow, and AC thermal effect [19], so the influence of these perturbing factors must be fully considered in the sorting process. At present, the research on the sorting of submicron particles by dielectrophoresis is still challenging. Dielectrophoresis can distinguish, to some extent, between particles with size differences of tens of nanometers. Some studies of dielectrophoresis have achieved a certain degree of high-resolution sorting of submicron particles by combining microfluidics, light-induced dielectrophoresis, and bias voltage [20,21,22]. Although these studies have improved the resolution of submicron particles to between about a few tens and a hundred nanometers, it is still difficult to realize the precision separation of submicron particles when the difference in particle size is within 50 nm or even less.

In this paper, we proposed a technique for multistage cyclic dielectrophoresis. Due to the small dielectrophoretic force on submicron particles, we designed and fabricated a face-to-face dielectrophoresis chip with an electrode spacing of 20 μm. In the multistage cyclic dielectrophoresis chip, particles were focused at the same initial position by the face-to-face focusing electrodes. After that, they were migrated a certain distance under the action of the sorting electrode, and the migration distance was positively correlated with the particle size. Finally, in order to realize the high-resolution sorting of particles under the perturbation factors of the flow and electric fields, we proposed the idea of multistage cyclic sorting, in which the effects of perturbation errors are greatly reduced by iterating the upper and lower limits of particle size through multistage cycling.

## 2. Materials and Methods

### 2.1. Multistage Cyclic Dielectrophoresis Sorting System

**Figure 1:**
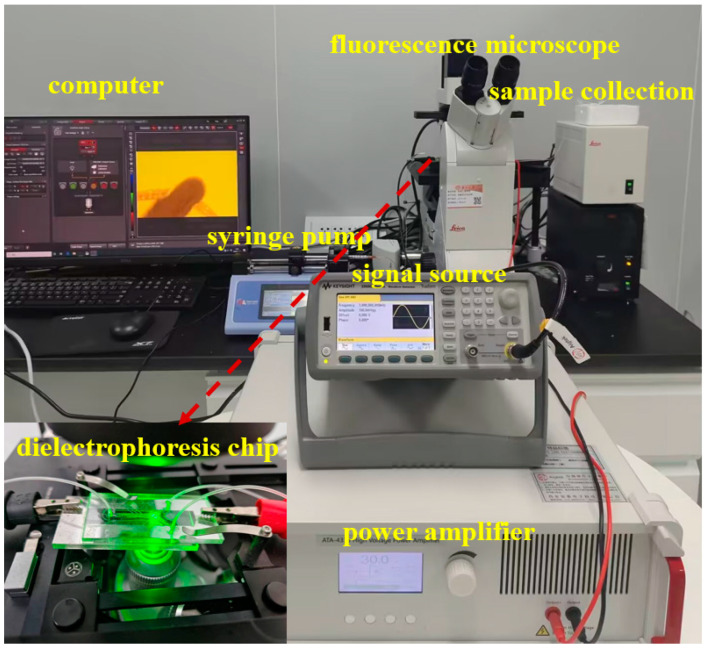
Photo of multistage circulating dielectrophoretic sieving system.

The experimental system for dielectrophoretic sorting of submicron microspheres is shown in Figure 1, which consists of four core components. Firstly, the high-frequency high-voltage electric field was generated by a signal generator (33521B, Agilent, Beijing, China) and a high-voltage power amplifier (ATA-4315, Aigtek, Xi’an, China) in concert. Secondly, a precision syringe pump (Pump 11 Pico Plus Elite, Friends Honesty Life Sciences Co., Ltd., Hong Kong, China) paired the system and precisely regulated the sample flow rate. Thirdly dynamic observation was realized by an inverted fluorescence microscope (DMi8, Leica Microsystems, Wetzlar, Germany). Finally, a nanoparticle tracking analyzer (Malvern Panalytical, Malvern, Britain) was utilized to characterize the samples. The experimental samples consisted of ultrapure water and fluorescent polystyrene microspheres, in which the ultra-pure water was prepared by the purification system (Cascada I, PALL, Beijing, China), and the fluorescent polystyrene microspheres (200 nm) were purchased from Yiyuan Biotechnology Co., Ltd. (Shanghai, China).

### 2.2. Materials and Structures of Dielectrophoretic Chips

In this study, a sandwich dielectrophoresis chip was designed, which consists of three parts: the top plate, the bottom plate, and the channel layer. The top and bottom plates were made of Polymethyl methacrylate(PMMA) sheets with a thickness of 3 mm, which were processed by laser cutting technology (Xintao Acrylic Co., Ltd., Shenzhen, China). The channel layer was made of polyethylene terephthalate (PET) film with a thickness of 20 μm, and the microchannel structure was cut using a laser (HZZ-V4000, HZZ EPHOTO Technology Co., Ltd., Guangzhou, China). Subsequently, the electrode patterns were cut out using a transparent tape with a thickness of 10 μm to serve as a mask for magnetron sputtering. Both the PET film and the transparent tape were purchased from DEYI Tape Co., Ltd. (Shanghai, China). An aluminum film was deposited on the surface of the top and bottom plates affixed with the electrode masks by magnetron sputtering (DentibE14, Kurt J. Lesker Company, Clairton, PA, USA). In addition, the magnetron sputtering parameters need to be optimized to ensure the quality of the metal film due to the poor adhesion of metal film on the PMMA substrate. After sputtering was completed, remove the transparent tape to obtain the aluminum electrode on the PMMA surface.

**Figure 2:**
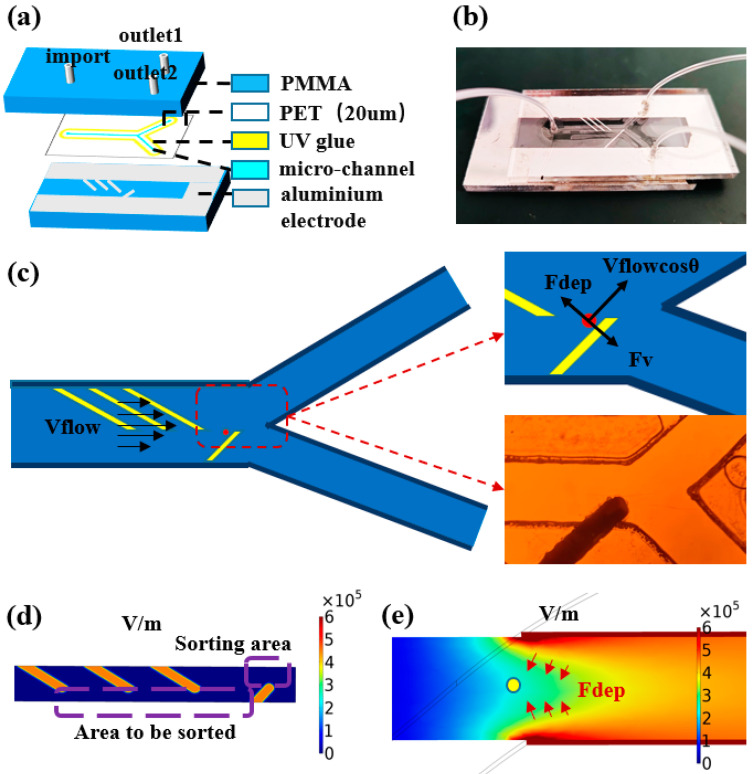
(**a**) Schematic material and structure of dielectrophoresis chip. (**b**) Photo of dielectrophoretic chip. (**c**) Schematic of forces on particles in the flow channel and photographs of the flow channel. (**d**) Simulation graphic of the area to be sorted and the sorting area. (**e**) Simulation graphic of the electric field distribution in the vertical direction.

As shown in the Figure 2a, sealing grooves are set around the microchannels in the chip design, and the UV curing adhesive (AA3492, LOCTITE, Shenzhen, China) is used to bond and seal the top plate, channel layer, and bottom plate. The amount of curing adhesive needs to be strictly controlled; too much will lead to channel blockage, while too little will affect the sealing effect. Finally, the PMMA top plate, PET channel layer, and PMMA bottom plate are precisely aligned to assemble a microchannel structure with top and bottom facing electrodes. After the chip is sealed, the height of the microchannel layer is 20 μm and the width is 500 μm. This face-to-face electrode design creates a symmetric three-dimensional electric field distribution on the *Z*-axis. The actual photo of the dielectrophoretic chip is shown in Figure 2b.

### 2.3. Multistage Cyclic Dielectrophoresis Sorting Mechanism

In an AC electric field, particles in an electrolyte solution are subjected to a variety of forces, including gravity, buoyancy, dielectrophoresis, viscous drag, AC electroosmosis, AC electrothermal, Brownian motion, and inter-particle interactions. Since the particles in this experiment are submicron particles, the effects of gravity and buoyancy are relatively minor and are therefore not discussed. However, unavoidable disturbing factors in the experiment, such as AC electroosmosis, AC electrothermal, and inter-particle interactions, can significantly affect the sorting process of the microspheres. In addition, fluid instability as well as adsorption and charge effects generated by the channel walls can introduce additional disturbances. Therefore, it is challenging to achieve higher particle size uniformity under these uncontrollable disturbances. In this experiment, the effect of these disturbing factors is effectively reduced by the method of dielectrophoretic force cyclic sorting.

As shown in Figure 2c, in the dielectrophoretic microfluidic chip, particles will pass sequentially through of three pairs of focusing electrodes and one pair of sorting electrodes. In this process, the motion of the particles is mainly affected by the following two forces: dielectrophoretic force (Fdep) and fluid drag force (Fdrag). Among these, the dielectrophoretic force is the force generated when the neutral particle is in a non-uniform electric field due to the difference between its dielectric properties and those of the surrounding medium. According to the relative magnitude of the polarizability of the particle and the medium, dielectrophoretic force can be divided into positive dielectrophoresis and negative dielectrophoresis: positive dielectrophoretic force drives the particle to move to the region where the gradient of the electric field strength increases; while negative dielectrophoretic force makes the particle migrate to the region where the gradient of the electric field strength decreases. For spherical electrolyte particles, the dielectrophoretic force on them can be described by the following expression:

(1) $$F_{dep}=2\pi r^{3}\varepsilon_{m}Re\left[K\left(\omega \right)\right]\nabla E^{2}$$

where r is the radius of the microsphere, ε_m_ is the dielectric constant of the suspension medium, K is the Clausius–Mossotti (CM) factor, and ω is the angular frequency of the electric field, and E denotes the localized electric field. K(ω) denotes the particle polarization rate, denoted as follows:

(2) $$K\left(\omega \right)=\frac{\varepsilon_{p}^{*}-\varepsilon_{m}^{*}}{\varepsilon_{p}^{*}+{2\varepsilon }_{m}^{*}}$$

where $\varepsilon_{p}^{*}$ and $\varepsilon_{m}^{*}$ are the complex permittivity of the particles and the medium, respectively. ε* = ε − jσ/ω, where ε is the permittivity, σ is the conductivity, and ω is the angular frequency. The real part of the frequency-dependent K(ω) determines the positive or negative dielectrophoretic force. When the polarizability of a particle is higher than the medium, i.e., Re[K(ω)] > 0, the particle is subjected to positive dielectrophoretic force and is attracted to the region with a high electric field; on the contrary, when the polarizability of a particle is lower than that of the medium, i.e., Re[K(ω)] < 0, the particle is subjected to negative dielectrophoretic force and is repelled outside the region of high electric field. In addition, the viscous resistance F_v_ can be expressed as follows:

(3) $$F_{v}=6\pi \mu rv$$

where μ denotes the viscosity of the solution, r denotes the particle radius, and v denotes the particle velocity (look on Solution as the frame of reference).

When a particle moves to the tilted electrode region, its motion behavior will be significantly affected by the particle initial velocity. Specifically, when the particle initial velocity exceeds the critical deflection velocity Vth, the particle will have enough kinetic energy to cross the potential barrier constituted by the tilted electrode pair; and when the particle initial velocity is lower than the critical value, the particle will be subjected to the action of the electric field force, and will undergo the deflection motion along the direction of the boundary of the tilted electrode. The Vth expression can be expressed as follows [23]:

(4) $$V_{th}=\frac{9\pi \varepsilon_{m}r^{2}Re\left[K\left(w\right)\right]V_{rms}^{2}}{64\mu H^{3}sin\theta }$$

In the theoretical model, H denotes the characteristic height of the microfluidic channel, V_rms_ is the rms voltage value of the applied AC electric field, and θ is the angle between the fluid flow direction and the electrode alignment direction.

When the particle motion velocity is lower than the critical velocity Vth, the particle will undergo electrophoretic deflection motion. In this state, the partial velocity of the particle along the flow direction can be expressed as Vflow*cosθ (where Vflow is the average flow velocity of the fluid), and the system reaches a mechanical equilibrium state, As shown in Figure 2c. This equilibrium state can be described by the following equation:

(5) $$6\pi \mu rvsin\theta =2\pi r^{3}\varepsilon_{m}Re\left[K\left(w\right)\right]\nabla E^{2}$$

In this process, particles with larger particle sizes are subjected to stronger negative dielectrophoretic forces and are pushed to the region away from the electrodes. As shown in Figure 2e, the use of face-to-face electrode configuration creates a symmetric electric field distribution in a vertical direction and generates a region of low electric field strength at the center height position of the channel. This electric field distribution characteristic is conducive to the aggregation of particles to the center of the channel height under the effect of negative dielectrophoretic force, thus ensuring a consistent initial spatial distribution of particles before sorting.

**Figure 3:**
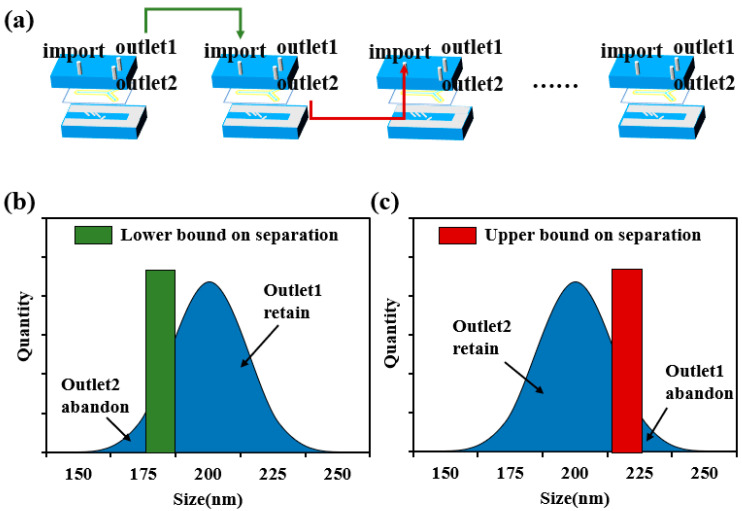
Schematic diagram of multistage cycle separation process: (**a**) demonstrates cyclic sorting of samples in a multistage dielectrophoretic chip; (**b**) particle size separation, retaining larger size particles; (**c**) particle size separation, retaining smaller size particles. The separation boundaries (green and red regions) in the figure have a certain width in order to characterize the error characteristics of the separation.

The sorting of submicron particles mainly depends on the dielectrophoretic force effect. Due to the size effect of the submicron particles, a higher electric field gradient is required to produce a significant dielectrophoretic force response. For this reason, a high-performance power amplifier is used in the experiment, which can generate distortion-free voltage signals up to 150 Vpp. In order to observe the particle sorting behavior in real time, the experimental system integrates an inverted fluorescence microscope. After the particles to be sorted are homogeneously mixed with ultrapure water to prepare a suspension, the sample is injected into the dielectrophoresis chip at a constant flow rate using a precision syringe pump. Considering that submicron particles are susceptible to random perturbations, such as Brownian motion, in the experimental environment, a multistage cyclic sorting strategy is innovatively adopted in this study to improve the sorting accuracy. As shown in Figure 2d, when the microspheres enter the microfluidic channel, they first pass through the three pairs of focusing electrodes and are aggregated in the area to be sorted. Subsequently, under the action of the sorting electrodes, the microspheres are separated into two groups according to the difference in particle size. By optimizing the applied voltage parameters, quantitative separation of the microspheres at the two outlets can be achieved. Theoretical analysis and preliminary experimental results show that the particle size is the key parameter to determine the deflection distance of the microspheres. Compared with microspheres with a smaller particle size, microspheres with a larger particle size have a higher probability to flow to outlet 1, thus realizing the lower limit of particle size sorting (i.e., excluding microspheres with smaller particle size with specific probability). Similarly, in the second stage sorting process, upper limit sorting (i.e., excluding microspheres with larger particle size with specific probability) can be realized by collecting microspheres at outlet 2. As shown in Figure 3b,c, the upper and lower limit sorting as a complete cycle, through multiple iterations can effectively reduce the impact of random perturbations, and ultimately realize the high-resolution screening of submicron particles, as shown in Figure 3a.

## 3. Results and Discussion

### 3.1. Submicron Microsphere Focusing

Given that submicron particles are susceptible to Brownian motion, AC electroosmosis, and AC electrothermal effect during the experimental process, the multistage cyclic dielectrophoresis (MC-DEP) technique is used in this study to effectively reduce the experimental errors. The working samples are prepared by diluting a suspension of fluorescent-labeled polystyrene microspheres (particle size of about 200 nm) with a mass fraction of 1% by 400 times in ultrapure water. Through systematic experiments, it is observed that, when the applied voltage frequency is in the range of 100 kHz to 1 MHz, the microspheres are subjected to strong negative dielectrophoretic force. It is worth noting that the optimization of experimental parameters is crucial: too low a voltage frequency can easily trigger solution electrolysis, while too high a voltage frequency may lead to signal distortion. In addition, when the applied voltage exceeds 60 Vpp, a significant electrolysis phenomenon (bubble generation) can be observed.

**Figure 4:**
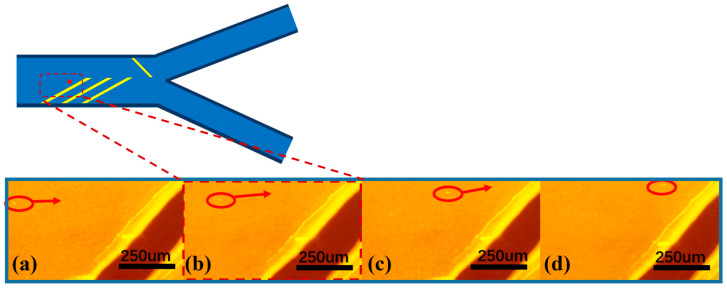
(**a**–**d**) demonstrate the particle migration time-series images captured by inverted fluorescence microscopy.

In the front region of the microfluidic channel, three pairs of focusing electrodes were symmetrically arranged at the top and bottom of the channel in order to realize the ideal microsphere focusing effect. The tilt angle of the focusing electrodes was set at 30–45° to achieve a better focusing effect, and the multi-electrode configuration can effectively enhance the aggregation effect of the microspheres and improve the spatial focusing accuracy. The motion trajectories of the microspheres are observed in real time by inverted fluorescence microscopy, and the sequential images in Figure 4a–d clearly demonstrate the dynamic aggregation process of the particles under the action of the dielectrophoretic force field. As shown in Figure 4, the particles gradually migrate to one side of the channel over time under the action of negative dielectrophoretic force. This process visually verifies the regulation of particle motion by dielectrophoretic force and provides an important basis for subsequent particle separation and manipulation. It is found that the flow rate parameter had a significant effect on the focusing effect: too high a flow rate would lead to a decrease in the microsphere focusing effect, while too low a flow rate can lead to a poor linear correlation between the particle migration distance in dielectrophoretic force and particle size, which further leads to an unstable separation effect. After parameter optimization, the syringe pump flow rate was set to 4 μL/min and the AC frequency was set to 1 MHz in this experiment. When the peak voltage is 30 Vpp, about 80% of the microspheres can be focused on the target area. When the voltage is increased to more than 40 Vpp, the focusing efficiency can reach more than 95%. The failure of some microspheres to achieve effective focusing may be attributed to the following factors: (1) non-uniform electric field distribution due to electrode processing defects; (2) non-specific adsorption and slippage phenomena of microspheres on the channel wall; (3) electroosmosis (AC electroosmosis), electrothermal effect, and electro-vortex flow [24] perturb the fluid field. These factors together affect the trajectory and focusing effect of the microspheres.

### 3.2. Submicron Microsphere Sorting

**Figure 5:**
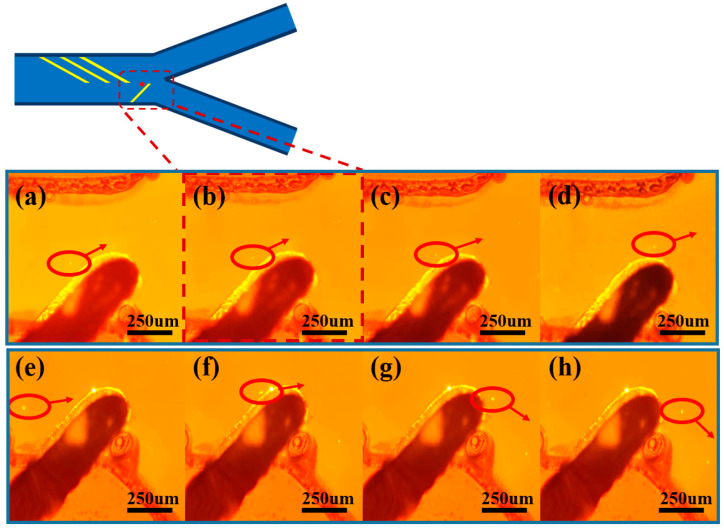
Time-series images of the motion of submicron microspheres under fluorescence microscopy, where (**a**–**d**) illustrate the trajectories of particles subjected to stronger dielectrophoretic forces flowing toward outlet 1, and (**e**–**h**) illustrate the trajectories of particles subjected to weaker dielectrophoretic forces flowing along the edges of the electrodes toward outlet 2.

As shown in Figure 5a–d, a pair of sorting electrodes with an inclination angle of 45° is configured in the downstream region of the microfluidic channel, and their tops are precisely positioned 20 μm below the center plane of the channel. When the population of microspheres enters the sorting region, microspheres with larger particle sizes are subjected to stronger negative dielectrophoretic forces and are unable to cross the potential barrier region formed by the electrode pair. These microspheres undergo a significant spatial shift as they approach the electrode tip, generating a migration of 40–100 μm in the vertical direction in the picture, and ultimately migrate in a directional manner to outlet 1. This physical process realizes the lower limit of particle size screening of the microsphere population, and the resulting microsphere ensemble has a larger average particle size.

After realizing the lower limit of particle size sorting of the microspheres, as shown in Figure 5e–h, microspheres with smaller particle sizes could not be effectively repelled to the watershed corresponding to outlet 1 when approaching the sorting region due to the relatively weak negative dielectrophoretic force. These microspheres migrate less than 20 μm under the dielectrophoretic force and flow along the edge of the electrode to outlet 2. In addition, experimental observations show that about 5–15% of the microspheres will directly cross the sorting electrode, and this part of the microspheres also flow to outlet 2. By collecting the microspheres at outlet 2, the upper limit of the microsphere particle-size screening can be realized, and the average size of the microsphere collection is reduced. By screening the upper and lower limits of particle size in three cycles, a highly homogeneous population of microspheres can be obtained with a significantly lower coefficient of variation of particle size.

**Figure 6:**
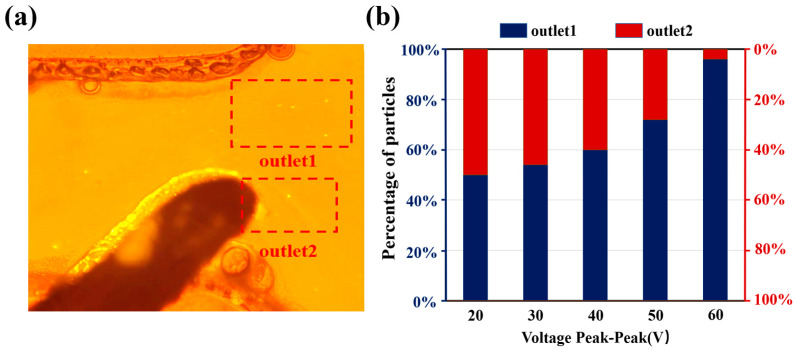
(**a**) Photograph of submicron particles flowing to the two outlets after passing through the separation electrode. (**b**) Ratio of microspheres for both outlets, voltage parameters (range: 20–60 Vpp, frequency 1 MHz, step: 10 Vpp).

The percentage of particles separated was observed dynamically by fluorescence microscopy, as shown in Figure 6a. Five replicate experiments were conducted at different time periods and 200 particles were recorded in each experiment. The experimental results showed that the error in the percentage of particles in the two exits was within 3%. The separation ratio at different voltages is shown in Figure 6b, the proportion of microspheres collected at outlet 1 show a significant growth trend with the increase of the applied voltage. When the voltage reaches 60 Vpp, more than 95% of the microspheres are sorted to outlet 1. But at this time, the electrode edge shows an obvious bubble generation phenomenon, indicating that a solution electrolysis reaction occurs. Based on the sorting mechanism, either too high or too low a separation ratio will lead to a decrease in resolution. In terms of the separation ratio, 40 Vpp to 50 Vpp is a more ideal operating voltage range. It is worth noting that too large a separation represents a larger microsphere loss rate. Under 40 Vpp voltage conditions, the microsphere separation ratio is about 6:4, and the microsphere recovery after sorting is about 1.4%; while, when the voltage is increased to 50 Vpp, the recovery decreased to 0.8%. In order to improve the overall recovery rate, the discarded microspheres during the sorting process are re-collected and subjected to secondary sorting, which could significantly improve the total recovery rate. After the dielectrophoretic sorting experiments were completed, the collected microspheres sizes were characterized using a nanoparticle tracking analyzer. Microsphere recovery can be calculated using the following equation:

(6) $$R_{p}=\prod_{i=1}^{n}R_{pi}$$

R_p_ denotes the particle recovery rate and n denotes the number of cycles.

### 3.3. Particle Size Optimization of Submicron Microspheres by MC-DEP

**Figure 7:**
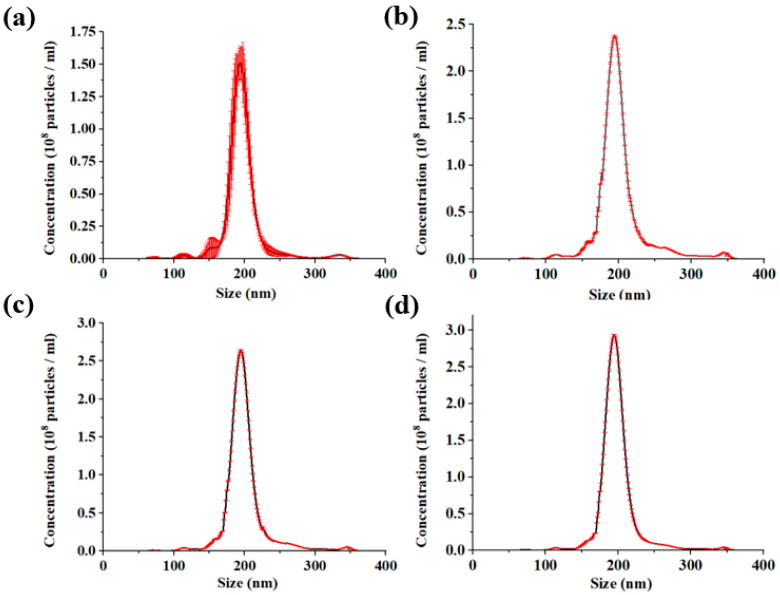
(**a**–**d**) show the particle size distribution of the initial sample, the first cycle, the second cycle, and the third cycle, respectively.

After completing the sub-micron microsphere sorting experiments, the microsphere particle size was characterized using a nanoparticle tracking analyzer. The particle size distribution is shown in Figure 7. The results show that the average diameter of the particles is 192.9 nm and the error before and after sorting is less than 1%. This shows that our circular sorting system does not cause excessive size shift. In addition, the standard deviation of particle size is 24.7 nm before sorting and 10.4 nm after three cycles of sorting. The concentration of the sample in the experiment was 5.21 × 10^9^ particles/mL. The particle size distribution obeys Gaussian distribution.

The coefficient of variation (CV value) of the particle size distribution is calculated based on the following formula:

(7) $$CV=\frac{\sigma }{D_{av}}\times 100\%$$

where CV denotes the coefficient of variation of particle size, σ denotes the standard deviation of particle size, and D_av_ denotes the average particle size.

In order to reduce the experimental chance error, the sorting experiments with different cycle times were repeated three times independently, and the fluctuation range of the CV value of the particle size was systematically counted. The experimental parameters were set as follows: the voltage conditions are 40 Vpp and 50 Vpp, and the frequency is fixed at 1 MHz. By comparing the changes of CV values under different parameter conditions, the effect of sorting and the homogeneity of particle size can be quantitatively evaluated.

**Figure 8:**
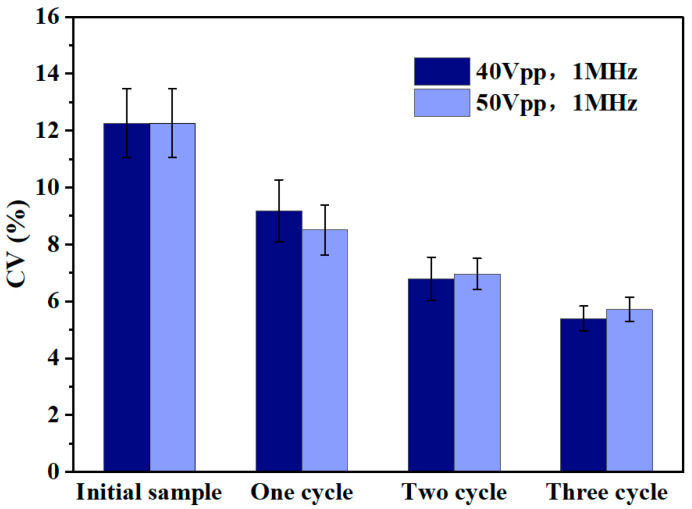
Statistical analysis of the coefficient of variation (CV) of particle size is carried out for sorting experiments with different number of cycles under the experimental conditions of voltage parameters of 40 Vpp and 50 Vpp, respectively, and a fixed frequency of 1 MHz.

As shown in Figure 8, the coefficient of variation (CV value) of the particle size of the polystyrene microspheres all show a significant reduction trend after three cycles of sorting (a total of six separation processes). It is noteworthy that the step-by-step sorting under the voltage condition of 40 Vpp not only obtained a smaller coefficient of variation (from 12.3% to 5.4%), but maintained a high recovery (about 1.4%). It was shown that, when the voltage is lower than 30 Vpp, the separation ratio of the microspheres at the two outlets is close to 1:1, at which time the perturbing factors, such as electroosmotic flow, electrothermal effect, and eddy current, became the main factors dominating the flow of microspheres, which led to a poor sorting effect. When the voltage exceeds 50 Vpp, most of the microspheres flow to outlet 1 under the dominant dielectrophoretic force, but there are still some microspheres that flow to outlet 2 under the influence of disturbing factors such as electric field defects, wall adsorption, and eddy currents, among other factors. Under this circumstance, it is difficult for the difference of the particle size to become the dominant factor determining the direction of the flow of the microspheres. Therefore, the selection of an appropriate operating voltage (40–50 Vpp range is recommended) is crucial for achieving efficient and accurate particle size sorting in the MC-DEP sorting process.

## 4. Conclusions

In view of the technical difficulties of high resolution sorting caused by small particle size and easy-to-be-disturbed errors, the multistage cyclic sorting strategy was innovatively proposed in this study, and the high-precision separation of submicron microspheres was successfully realized. The throughput of the sorting process is 2.08 × 10^7^ particles/min. Due to the molecular interaction forces during dielectrophoretic sorting, the concentration values of particles in the sample should be less than 2 × 10^10^ particles/mL.

Our research group designed and prepared a face-to-face dielectrophoresis chip, which effectively eliminates the longitudinal difference in the microsphere sorting process, and improves the accuracy of sorting compared with the traditional planar electrode structure. Through systematic experimental studies, we found that the submicron microspheres all exhibited significant negative dielectrophoresis effects when the voltage frequency is in the range of 100 kHz to 1 MHz. Specifically, when the voltage is lower than 30 Vpp, the dielectrophoretic force effect is not obvious. When the voltage reaches 60 Vpp, the electrolysis phenomenon occurs. The optimization experiments show that both voltage conditions of 40 Vpp and 50 Vpp can significantly reduce the particle size variation coefficient of submicron microspheres. Under the optimal parameter conditions (flow rate: 4 μL/min, voltage: 40 Vpp, frequency: 1 MHz), the particle size variation coefficient of submicron microspheres is significantly reduced from the initial 12.3% to 5.4%.

This study provides a new technical idea and method for accurate sieving of submicron-scale particles. However, the processing throughput of the system is relatively small and requires some manual operation. Further research can be optimized in the following aspects: (1) improving the design of the chip structure to increase the sorting efficiency; (2) realizing the integration and automation of the chip functions; and (3) establishing the quality control standards for recycled particles. In the future, we will deeply analyze the various disturbance errors suffered by submicron microspheres in dielectric electrophoresis chips to further improve the accuracy and reliability of separation. Further, the dielectrophoresis chip may be integrated into a microfluidic chip. Examples include microfluidic chips for preparing photonic crystal films, microfluidic chips for tumor marker identification.

## Data Availability

The datasets generated during and/or analyzed during the current study are available from the corresponding author on reasonable request.
